# Protein Profiling of Wild-Caught *Phlebotomus papatasi* in Morocco: First Observation of Nematodes in Moroccan Population of Sandflies

**DOI:** 10.3390/pathogens14101012

**Published:** 2025-10-07

**Authors:** Mohamed Daoudi, Myriam Beaulieu, George Dong, Momar Ndao, Samia Boussaa, Mohamed Hafidi, Ali Boumezzough, Martin Olivier

**Affiliations:** 1 Infectious Diseases and Immunity in Global Heath Program, Research Institute of the McGill University Health Centre, Montreal, QC H4A 3J1, Canada; mohamed.daoudi@mail.mcgill.ca (M.D.); myriam.beaulieu2@mail.mcgill.ca (M.B.); george.dong@mail.mcgill.ca (G.D.); momar.ndao@mcgill.ca (M.N.); 2 Department of Medicine, Microbiology and Immunology, McGill University, Montreal, QC H3A 2B4, Canada; 3 Division of Experimental Medicine, McGill University, Montreal, QC H3A 2B4, Canada; 4 National Reference Centre for Parasitology, Research Institute of the McGill University Health Centre, Montreal, QC H4A 3J1, Canada; 5 Higher Institute of Nursing Professions and Health Techniques, Ministry of Health and Social Protection, Rabat 10020, Morocco; samiaboussaa@gmail.com; 6 Microbial Biotechnologies, Agrosciences and Environment Laboratory, Faculty of Sciences Semlalia, Cadi Ayyad University, Marrakesh 40080, Morocco; hafidi@uca.ac.ma (M.H.); aboumezzough@gmail.com (A.B.)

**Keywords:** pathogens circulation, *Phlebotomus papatasi*, Morocco

## Abstract

Phlebotomine-borne diseases, transmitted by sand flies, cause significant public health burdens worldwide. In Morocco, *Phlebotomus papatasi* is a primary vector for *Leishmania major* and phleboviruses. Despite extensive research in other countries, entomopathogenic parasite investigations in *P. papatasi* have not been conducted in Morocco until now. This study performed proteomic analysis of female *P. papatasi* collected from four Moroccan localities using liquid chromatography-tandem mass spectrometry (LC-MS/MS). Our analysis revealed that *Phlebotomus papatasi* peptides were the most abundant, with 884 peptides identified. Additionally, we detected 732 peptides from nematodes, 86 from *Leishmania major*, 79 from *L. infantum*, eight from *L. tropica*, and two peptides associated with phleboviruses. Microscopic examination of 1752 sand flies confirmed *P. sergenti* female infected with Tetranematidae, *Didilia* spp. in Imintanout (Z2). This study provides the first report of nematodes in sand flies in Africa and represents the first application of proteomics to identify pathogens carried by *P. papatasi*. These findings highlight remarkable proteomic differences among localities and generate critical data for understanding parasite-vector interactions.

## 1. Introduction

Phlebotomine sandflies (*Diptera: Psychodidae*, Phlebotominae) are small, fragile, blood-sucking insects with a wide range of hosts, facilitating pathogen transmission to humans and other animals [1,2]. These insects are recognized as vectors of various diseases, including canine and human leishmaniasis, bartonellosis, and several arboviruses [3]. Leishmaniasis is a significant public health concern in Morocco, with a broad geographical distribution of sand fly species [4]. Moroccan sand fly populations act as vectors not only for protozoa but also for viruses [5].

*Phlebotomus papatasi* is widely distributed around the Mediterranean basin [6]. This species is quite common and has a significant ecological plasticity [7,8]. The wide distribution of *P. papatasi* extends across North Africa, through Eurasia, and into India [9]. It is the vector responsible for transmitting cutaneous leishmaniasis (CL) and sandfly fever that occurs in the Mediterranean regions [10]. Many sandfly-borne phleboviruses such as the Toscana virus, Sicilian and Naples viruses are also transmitted to humans by *P. papatasi* in countries around the Mediterranean Sea and eastwards to central Asia and India [11]. Aside from its vectorial role in human and animal diseases, *P. papatasi* has also been observed to have an entomophilic nematode infestation in Pondicherry, India [12]. In Morocco, *P. papatasi* is the primary vector of zoonotic leishmaniasis, the most prevalent form of the disease in terms of case numbers [13]. This species is associated with all *L. major*-endemic cutaneous leishmaniasis (CL) foci across the country, particularly in arid regions [8]. *P. papatasi* exhibits peak activity during the hot, dry season and is most abundant when ambient temperatures range between 32 and 36 °C [13]. It has demonstrated a strong adaptation to arid climatic conditions [14,15].

Given the complex role *P. papatasi* plays in pathogen transmission, advanced molecular techniques such as metabarcoding and metagenomics are essential for further understanding the pathogens circulating in these sandfly populations. These techniques are particularly effective for determining the whole genome sequences of pathogens [16,17]. In parallel, liquid chromatography coupled with tandem mass spectrometry (LC-MS/MS) has become a powerful approach for studying host–pathogen interactions at the protein level [18]. Label-free LC-MS/MS allows the identification and quantification of thousands of proteins across multiple samples in a single run, providing an unprecedented opportunity to explore proteomic profiles and their changes in response to biological challenges [19].

In the present study, we analyzed female *P. papatasi* from four Moroccan populations, using LC-MS/MS to identify circulating pathogens. This proteomic analysis provided rich data, revealing peptides and their corresponding proteins, contributing valuable information to the understanding of this species in Morocco.

## 2. Materials and Methods

### 2.1. Sand Fly Collection and Species Identification

Sandflies were collected using miniature CDC light traps (John W. Hook Co., Gainesville, FL, USA) between June 2018 and June 2019 from four localities in Morocco; three of them are endemic foci of leishmaniasis (Z1: Errachidia (31°56′52.6″ N 4°25′47.7″ W), Z2: Imintanout (31°10′18.1″ N 8°51′02.4″ W), Z3: Zagora (30°20′52.5″ N 5°50′13.1″ W)) and one is a non-endemic foci from NE: Marrakech (31°39′11.6″ N 8°01′30.2″ W) [18]. A total of 1752 sandflies were collected from the four localities investigated. Male and female sandflies were separated using a cold table in the laboratory and stored in pools of 50 specimens at −80 °C in the Microbial Biotechnologies, Agrosciences and Environment Laboratory in Marrakech, Morocco, until use.

Since the type of trap can substantially affect the quality of protein spectra in collected sandflies, we used specimens trapped using CDC techniques to provide a more accurate proteomic analysis, contrary to sticky trap collection techniques which could have compromised the samples and their proteomic analysis accuracy [20].

Females were washed carefully using PBS solution and then dissected and identified individually at the species level according to morphological characteristics. The genitalia and head of the female sandflies were mounted on a slide and species identification was made using identification keys [21]. The thorax and abdomen of female *P. papatasi* were preserved in RNAlater solution for use in the proteomic assay.

### 2.2. Sample Preparation and Liquid Chromatography–Ms/Ms

Proteomic analysis by LC-MS/MS was performed at the proteomic facility of the Research Institute of the McGill University Health Centre (RI-MUHC; Montréal, QC, Canada). From each locality, fifteen *P. papatasi* females (arranged in three pools of five specimens) were analyzed to detect proteins linked to medically important pathogens, including *Leishmania* spp., phleboviruses, and nematodes.

The sandflies collected were preserved in 70% ethanol and transported to the RI-MUHC. The remaining sandflies were examined under a binocular microscope for the presence of potential nematodes. The infected specimens were dissected to remove the nematodes from the sand fly body and identified morphologically according to the Moroccan sand fly key [21]. Nematodes were identified by comparing the body size, egg diameter, and morphological characteristics with those in previous studies of the Tetradonematid nematodes [22,23,24]. The sandflies collected were preserved in 70% ethanol and transported to the RI-MUHC.

A standard TCA protein precipitation was first performed to remove ethanol from *P. papatasi* specimens. Protein extracts were then re-solubilized in 10 µL of a 6M urea buffer. Proteins were reduced by adding 2.5 µL of the reduction buffer (45 mM DTT, 100 mM ammonium bicarbonate) for 30 min at 37 °C and then alkylated by adding 2.5 µL of the alkylation buffer (100 mM iodoacetamide, 100 mM ammonium bicarbonate) for 20 min at 24 °C in the dark. Prior to trypsin digestion, 20 µL of de-ionized distilled water was added to reduce the urea concentration to 2M. A total of 10 µL of the trypsin solution (5 ng/µL of trypsin sequencing grade from Promega, 50 mM ammonium bicarbonate) was added to each sample. Protein digestion was performed at 37 °C for 18 h and stopped with 5 µL of 5% formic acid. Protein digests were dried in vacuum centrifuge and stored at −20 °C until LC-MS/MS analysis.

Liquid chromatography-tandem mass spectrometry (LC-MS/MS) was performed at the RI-MUHC proteomic facility as described by Atayde et al. [18]. Sample proteins were precipitated with 15% trichloroacetic acid (TCA)/acetone and digested with trypsin at a final concentration of 2 ng/mL. After an 18 h incubation at 37 °C, the reactions were quenched by the addition of formic acid to a final concentration of 1% prior to the LC-MS/MS analysis. The LC column was a PicoFrit fused silica capillary column (New Objective, MA, USA) self-packed with C-18 reverse-phase material (Phenomenex, CA, USA). This column was installed on the Easy-nLC II system (Proxeon Biosystems, Odense, Denmark) and coupled to the Q Exactive mass spectrometer (Thermo Fisher Scientific, MA, USA) equipped with a Proxeon nanoelectrospray Flex ion source. The buffers used for chromatography were 0.2% formic acid (buffer A) and 100% acetonitrile/0.2% formic acid (buffer B). Peptides were loaded onto a column at a flow rate of 600 nL/min and eluted using a two-slope gradient at 250 nL/min. Solvent B first increased from 2% to 40% in 85 min and then from 40% to 80% in 25 min. LC-MS/MS data were acquired using a data-dependent top-15 method, with standard settings applied for all mass spectrometer parameters [18].

### 2.3. Protein Database Search

The peak list files were generated using Proteome Discoverer (version 2.1) using the following parameters: minimum mass set to 500 Da, maximum mass set to 6000 Da, no grouping of MS/MS spectra, precursor charge set to auto, and minimum number of fragment ions set to five. Protein database searching was performed with Mascot 2.6 (Matrix Science, IL, USA) against the Moroccan *Leishmania* species (*L. major*, *L. infantum* and *L. tropica*), Phleboviruses, Nematode protein databases. The mass tolerances for precursor and fragment ions were set to 10 ppm and 0.1 Da, respectively. Trypsin was used as an enzyme allowing for up to 1 missed cleavage. Cysteine carbamidomethylation was specified as a fixed modification and methionine oxidation as variable modifications. Data analysis was performed using Scaffold (version 4.10.0). Only proteins with a minimum of three peptides and peptides scoring higher than 20 were considered. To assess differential protein expression in *Phlebotomus papatasi*, we established a synthetic control based on sandfly-specific proteins, which served as a baseline reference. In standard proteomic analyses, volcano plots are used to compare the same set of proteins across two conditions, with log_2_; fold change representing the magnitude of change and *p*-values indicating statistical significance. In contrast, our study examined different protein sets, including pathogen-related proteins (from *Leishmania*, nematodes, and Phlebovirus) as well as intrinsic sandfly proteins. The use of synthetic control relies on the assumption that sandfly-specific proteins remain relatively stable across samples, since their expression is not expected to be substantially affected by infection.

## 3. Results

The analysis of three pools of five *P. papatasi* specimens from each of the four localities enabled differentiation of biomarker peptides among the sandfly populations, including those associated with *Leishmania* spp., phleboviruses, and nematodes. We compared the list of identified proteins across the localities and observed that most protein hits were consistent across all locations. Detailed spectra, peptide reports, and sample replicates from each locality are available in Supplementary Material File S1. We identified varying levels of abundance for *P. papatasi* proteins, including spot number, UniProt identifier, protein name, and function, as annotated through Blast2GO (version 6.0.3) similarity searches (refer to Supplementary Material File S1).

**Figure 1:**
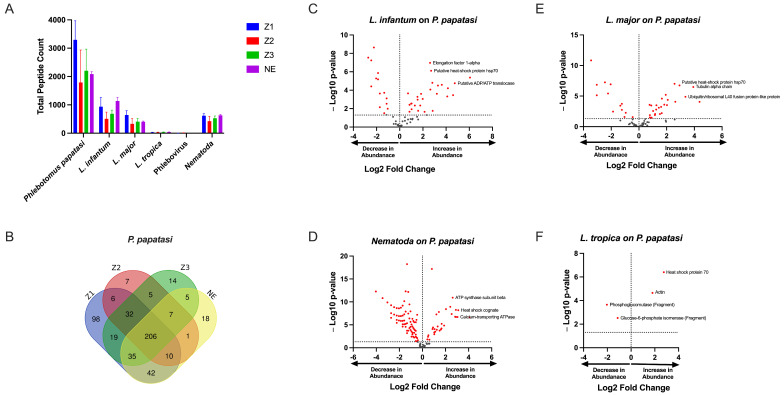
Protein Content Analysis of *P. papatasi* from Four Moroccan Localities. (**A**) Total number of identified peptides in *P. papatasi* samples across four Moroccan localities. (**B**) Venn diagram illustrates the shared and unique peptide among *P. papatasi* populations from the studied localities. (**C**–**F**) Volcano plots displaying the differential abundance of identified pathogen-associated peptides compared to *P. papatasi* proteins. Locality abbreviations: Z1, Errachidia; Z2, Imintanout; Z3, Zagora; NE, Marrakech.

Our initial analysis clearly showed that *P. papatasi* peptides were the most abundant, with 884 peptides identified, which was expected. This was followed by 732 peptides from nematodes, 86 from *Leishmania major*, 79 from *L. infantum*, eight peptides from *L. tropica*, and two peptides associated with phleboviruses (Figure 1A). The Venn diagram illustrates the distribution of shared and unique proteins among *P. papatasi* populations from the four studied localities, revealing a core set of 206 peptides common to all specimens. This shared peptide set suggests a conserved protein profile across the different geographical populations. Additionally, we observed unique proteins specific to each region, indicating potential location-dependent variations. These unique peptides may reflect regional environmental influences, genetic adaptations, or differences in pathogen exposure among sandfly populations (Figure 1B).

By comparing spectrum or peptide counts of different samples against this synthetic control, we identified peptides whose expression levels were significantly altered in response to infection. This approach effectively distinguishes infection-driven variations from the inherent expression of sandfly proteins. The results highlight key proteins associated with pathogen presence, providing insights into host–pathogen interactions within different sandflies (Figure 1C–F).

**Figure 2:**
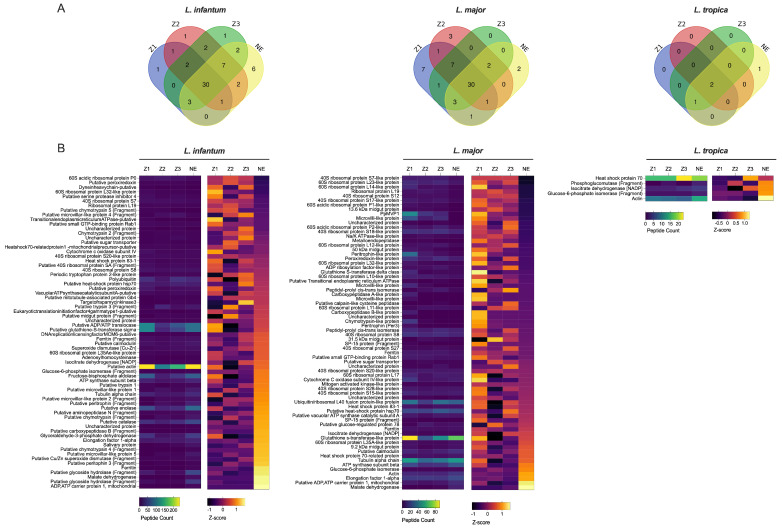
Quantitative Proteomic Profiles of *P. papatasi* for *Leishmania* Across Different Moroccan Localities. (**A**) Relative expression levels of *Leishmania* species proteins obtained from mass spectrometry analysis (*L. major*, *L. infantum*, *L. tropica* databases) using biological triplicates for each locality (*n* = 3) and analyzed with Mascot (version 2.6) and Scaffold (version 4.8) software. (**B**) Heat map representing quantitative proteomic profiles, generated using Z-scores without normalization and clustered based on protein sets with positive Z-scores. Locality abbreviations: Z1, Errachidia; Z2, Imintanout; Z3, Zagora; NE, Marrakech.

*Leishmania* spp. peptides were present in different localities including the non-endemic locality (NE) (Figure 1A). Comparing spectral counts for *Leishmania* spp. peptides from the different localities studied, our results show a significant difference for *L. tropica* proteins, but not for other *Leishmania* spp. by peptide group-based spectral count differentiation (Figure 2). We describe *Leishmania* species determination on entomological samples based on partial sequencing of *Leishmania* spp. proteins such as the heat-shock protein 70 gene (Hsp70), Alpha tubulin protein (Tuba1) and Ubiquitin (Ubi) (Figure 2). These proteins involve a variety of cellular functions for *Leishmania* spp. We observed the presence of *Leishmania* spp. biomarker proteins in *P. papatasi* at all sampled sites, including the non-endemic area (NE) (Figure 2). A noteworthy protein detected was the viscerotropic leishmaniasis protein specific to *L. tropica*, which is responsible for anthroponotic cutaneous leishmaniasis in Morocco [18]. The clustering of differentially identified proteins for *L. tropica*, *L. major*, and *L. infantum* is presented in Figure 2. We also observed a shared abundance of Hsp70, Tuba1, and Ubi peptides across all three Leishmania species, while other peptides showed differential abundance or were absent in each species. Mitochondrial isocitrate dehydrogenase, partial aconitase, and vacuolar ATP synthase catalytic subunit A (putative) were the main peptides highlighting *Leishmania tropica* specifically in this analysis, whereas hypothetical protein (conserved) and elongation factor 1-alpha were not detected in this species but were shared between *L. infantum* and *L. major*. In addition, the putative calmodulin peptide appears to be specific for *L. major*. The heat-shock 70-related protein 1 (mitochondrial precursor, putative) was highly detected in *L. major*, but not in the other *Leishmania* species analyzed.

Regarding phleboviruses, the number of specific proteins according to locality is lower, as these did not show in abundance. We recorded only two phlebovirus peptides that refer to Sand fly fever virus (SFSV) and Severe fever with Syndrome Virus (SFTSV) (Figure 1A).

**Figure 3:**
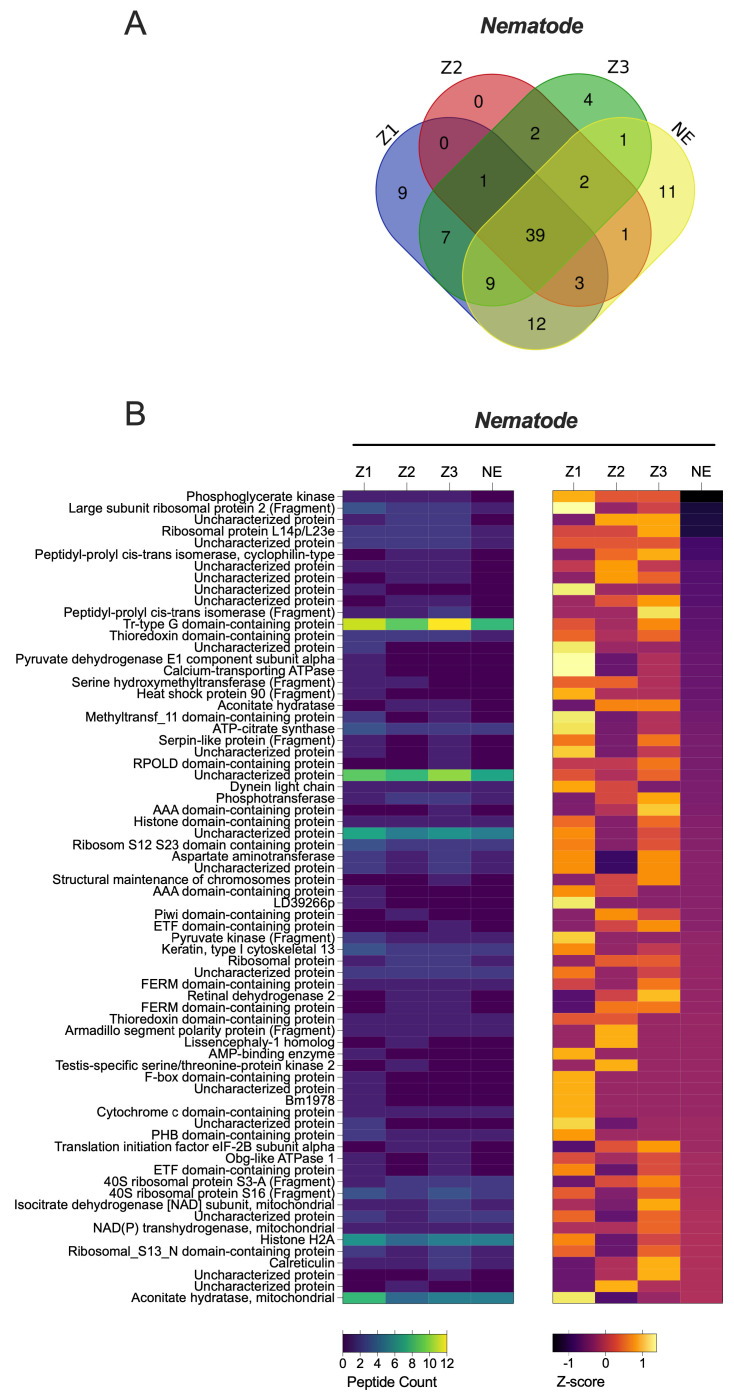
Quantitative Proteomic Profiles of *P. papatasi* for Nematodes Across Different Moroccan Localities. (**A**) Relative expression levels of Nematodes proteins obtained from mass spectrometry analysis using biological triplicates for each locality (*n* = 3), analyzed with Mascot and Scaffold Software. (**B**) Heat map representing quantitative proteomic profiles, generated using Z-scores without normalization, and clustered based on protein sets with positive Z-scores. abbreviations: Z1, Errachidia; Z2, Imintanout; Z3, Zagora; NE, Marrakech.

Interestingly, nematode proteins were consistently found to be highly abundant in sandflies across all localities, as indicated by the high number of unique peptides detected (Figure 1A and Figure 3, Supplementary Material File S2). This observation suggests the potential value of conducting direct microscopic examinations of sandflies collected from different localities to further explore the presence of nematodes.

**Figure 4:**
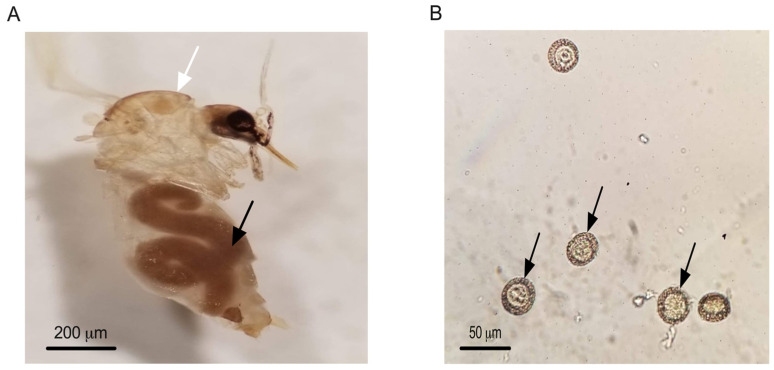
Female of *P. sergenti* from Imintanout locality infected with a single female. Tetranematidae. Black arrow pointing to Tetranematidae, white arrow pointing to *P. sergenti* (**A**) Eggs of a Tetradonematidae isolated from *P. sergenti* in Morocco. Arrows pointing to Tetradonematidae eggs (**B**).

Direct microscopic examination of 1752 sand fly specimens collected from multiple localities revealed nematode infection in only one specimen, which was found in the Imintanout locality (Z2) in central Morocco. Morphological analysis identified this specimen as a female *Phlebotomus sergenti*. Based on measurements of the nematodes’ body length (approximately 3400 μm), body width (around 200 μm), and egg diameter (26.4 ± 2.2 μm), the nematodes were identified as *Didilia* species belonging to the family Tetradonematidae (Figure 4).

## 4. Discussion

To better understand the potential pathogens circulating in *Phlebotomus papatasi* populations in Morocco, we conducted a proteomic analysis to detect *Leishmania* spp., phleboviruses, and entomopathogenic parasites across different study localities. The application of proteomics in the surveillance of vector-borne pathogens in natural sandfly populations remains relatively unexplored, making this study one of the few to leverage this approach for pathogen detection in sandflies. Among the 22 recorded sandfly species in Morocco, five species of the genus *Phlebotomus* have been identified as vectors of the three nosogeographic forms of leishmaniasis present in the country. Specifically, *Phlebotomus papatasi* is a proven vector of *L. major*, the causative agent of zoonotic cutaneous leishmaniasis [25]. Our findings contribute to a better understanding of the natural circulation of pathogens within sandfly populations and highlight the potential of proteomic tools in vector-borne disease surveillance. *P. ariasi*, *P. longicuspis*, and *P. perniciosus* are vectors of *L. infantum* [26], while *P. sergenti* is a proven vector of *L. tropica* [27]. Proteomics, particularly LC-MS/MS, may improve our understanding of parasite biology and pathogenesis and has also paved the way for the screening of pathogens proteins in *Phlebotomus papatasi*. Our results revealed that some specific biomarker proteins such as Hsp-70, alpha-tubulin and ubiquitin of *L. infantum* and *L. tropica* proteins were present in *P. papatasi*. Our emphasis on Hsp70, Tuba1, and Ubi arises from their well-documented roles as central regulators of *Leishmania* biology that directly influence the parasite’s capacity to persist within the sandfly and establish infection in the mammalian host. Heat shock proteins such as Hsp70 have been shown to form stage-specific phosphorylation-dependent complexes that buffer the parasite against thermal and oxidative stresses encountered during vector colonization, while simultaneously facilitating the differentiation of promastigotes into the infectious metacyclic stage [28]. The parasite cytoskeleton, in turn, undergoes significant remodeling during development in the vector, and recent work has demonstrated that post-translational modifications of alpha-tubulin (Tuba1), including detyrosination, are critical for shaping cell morphology, maintaining flagellar function, and promoting efficient motility and virulence [29]. Complementing these processes, ubiquitin-mediated protein conjugation has emerged as a key mechanism controlling proteome plasticity, with UBC2-UEV1-dependent ubiquitination shown to be essential for differentiation between life-cycle stages [30]. Together, these proteins highlight the interconnected pathways of stress adaptation, cytoskeletal remodeling, and regulated protein turnover that underlie parasite development within the sandfly midgut, reinforcing their biological relevance in the context of host–pathogen interactions. Among significant proteins which are different in our analysis for *Leishmania* species, we could mention hypothetical protein-conserved, elongation factor 1-alpha and Vacuolar ATP synthase catalytic subunit A-putative. Those peptides play a crucial role in oxidative stress defense and protein synthesis [31]. We report in our analysis the abundance of these proteins, or *L. major* and *L. infantum*, and the absence of *L. tropica*. The Calmodulin protein is a calcium-binding protein and one of the cell-signaling molecules in *Leishmania* species [32,33]; however, it was detected only in *L. infantum* and not in the other *Leishmania* species. Among the specific mitochondrial proteins identified that highlight species specificity, mitochondrial isocitrate dehydrogenase and aconitase were found in *L. tropica* but absent in other *Leishmania* species. These proteins are essential for parasite proliferation and participate in critical cellular functions, including transport, stress response, and signaling [34]. These proteins have been investigated to permit the discrimination of medically important *Leishmania* species worldwide without the need for parasite isolation [19,35,36,37,38]. Among them, some proteins that are highly conserved along the eukaryotic evolutionary tree are available on the genome sequences for several *Leishmania* species and are important in genome organization of *Leishmania* species [39,40]. The presence of all Moroccan *Leishmania* nosogeographic species proteins in *P. papatasi* could be explained by the overlapping of three *Leishmania* spp. in Morocco, which renders the Moroccan leishmaniasis epidemiological profile as inaccurate. Effectively, *L. tropica* has been recorded in endemic *L. major* foci [41,42], and visceral (VL) leishmaniasis cases have been found in established foci of zoonotic cutaneous leishmaniasis ZCL in Morocco [43]. The presence of viscerotropic *L. tropica* antigen protein could stem from the presence of zymodeme (*L. tropica*-279) which is responsible for canine VL in Morocco [44]. Since there is no evidence that *P. papatasi* is involved in the transmission of any *Leishmania* species other than *L. major* [44], several experimental studies demonstrated that *P. papatasi* feeding on lesions or through a membrane will support the full growth and development of *L. major*, but not of any other *Leishmania* species [45,46]. They noted a high resistance of sand-fly vectors to various species of *Leishmania* with an increase in ingested parasites [45,47].

Moroccan populations of sandflies are not only vectors of protozoa, but also viruses. Es-Sette et al. [27] identified the Toscana virus and its distribution in our country [5]. In central Morocco, antibodies against the Naples virus and Sicilian virus have been observed in human populations living in this part of Morocco [11]. Interestingly, antibodies of these two viruses were detected in areas where *P. papatasi* is present and abundant [48], raising the question of the potential incrimination of *P. papatasi* in the transmission of those viruses. Seemingly, our proteomic analysis for phlebovirus detection revealed a low number of identified peptides; for example, the polymerase SFSV protein was recorded in Zagora (Z3) and Marrakech (NE). Also, some emerging tick-borne viruses [49,50,51] such as SFTSV glycoprotein detected in Imintanout (Z2), Zagora (Z3) and Marrakech (NE) and Errachidia (Z1), Zagora (Z3) and Marrakech (NE) for UUKV nucleocapsid protein were identified. Lower numbers of identified peptides could be due to RNA degradation, while *P. papatasi* morphological identification was performed prior to proteomics analysis [52].

Unlike our findings in the phlebovirus proteomic screening, a substantial number of identified proteins indicated the presence of nematodes (Figure 1). This important discovery prompted us to conduct microscopic examinations of *Phlebotomus papatasi* and other sand fly species collected from various localities. Entomoparasitic nematodes of phlebotomine sand flies have been reported in several regions, including Pakistan, Saudi Arabia, Afghanistan, and Portugal [22,53,54,55,56,57,58], but until now, they have not been observed in Africa. Notably, a Tetradonematidae nematode was reported infecting *Lutzomyia longipalpis* in a laboratory colony maintained at the National Institute of Health in Bogotá, Colombia, and was also detected in *P. sergenti* and *P. ariasi* in Portugal [55,56]. In our study, we examined 1752 sand fly specimens from four localities for nematode presence. One female *P. sergenti* specimen was found infected at the Imintanout locality, a known endemic focus for cutaneous leishmaniasis caused by *Leishmania tropica* in Morocco. Morphological analysis identified the nematodes as *Didilia* spp. (Tetradonematidae) [22,23,24]. This finding represents the first report of *Didilia* spp. in sand flies from North Africa and indeed from the African continent. A limitation of our study is the lack of molecular identification of the nematodes detected in *P. sergenti*.

While proteomic and microscopic analyses provided strong evidence for the presence of nematodes, molecular confirmation would be necessary to validate species identity and to further explore their epidemiological significance.

## 5. Conclusions

This study represents the first application of LC-MS/MS-based proteomics to identify pathogen-related proteins in wild-caught *Phlebotomus papatasi* from Morocco. Proteins associated with *Leishmania*, phleboviruses, and nematodes were detected, providing molecular evidence of co-circulating pathogens in sand fly populations. Additionally, microscopic analysis revealed the presence of Tetranematidae, *Didilia* spp. in the same region endemic for leishmaniasis, marking the first report of nematode infections in sand flies from the African continent.

These findings highlight the complex pathogen diversity harbored by Moroccan sand fly populations and underscore the need for further investigation into their potential role as vectors of multiple parasites beyond *Leishmania*.

## Data Availability

All required data are available in the manuscript. Any additional data can be provided upon request.

## Supplementary Materials

The following supporting information can be downloaded at: https://www.mdpi.com/article/10.3390/pathogens14101012/s1, File S1: Detailed spectra, peptide reports, and sample replicates from each locality; File S2: UniProt analysis results.
